# Supplementary material for: Analysis of PPARγ Signaling Activity in Psoriasis
Source: Int J Mol Sci. 2021 Aug 10;22(16):8603. doi: 10.3390/ijms22168603 (PMC8395241; doi:10.3390/ijms22168603)
Supplement: Supplementary file 1 [file ijms-22-08603-s001.zip › Supplemental materials_Analysis of PPARg signaling activity in psoriasis/Pathway models/Models images and html files/Differentiation of psoriatic T cells/190024.html]

keratinocyte proliferation


# Cell Process keratinocyte proliferation

|  |  |
| --- | --- |
| URN | urn:agi-gocellproc:0043616 |
| Total Entities | 0 |
| Connectivity | 1046 |
| Name | keratinocyte proliferation |

---

|  |  |
| --- | --- |
| Pathway | Gap Junction Assembly |
|  | Hair Follicle Keratinocyte Apoptosis |
|  | IL17 Signaling in Psoriasis |
|  | T-Cells Differentiation Block in Psoriasis |
|  | IL36 Receptor Antagonist (IL1F5) Deficiency (DITRA) |
|  | EGF/cyclinD1 Signaling in Acquired Middle Ear Cholesteatoma |
|  | Cholesteatoma |
|  | Dioxin Induced Chloracne (Hypothesis) |
|  | PPAR Psoriasis |
|  | PPARG inhibits phenotypes assotiated with psoriasis |
|  | efects selected |
|  | Neighbors of keratinocyte proliferation |
|  | 1\_Differentiation of psoriatic T cells |
|  | 2\_Interleukin-17 and interleukin-22 signaling in psoriasis |
|  | Differentiation of psoriatic T cells |
|  | Interleukin-17 and interleukin-22 signaling in psoriasis |

---

|  |  |
| --- | --- |
| MedScan ID | 4143616 |

---

|  |  |
| --- | --- |
| Alias | keratinocyte proliferation |

---

|  |  |
| --- | --- |
| GO ID | 0043616 |

---
